# Free of acrylamide sodium dodecyl sulphate (SDS)‐based tissue clearing (FASTClear): a novel protocol of tissue clearing for three‐dimensional visualization of human brain tissues

**DOI:** 10.1111/nan.12361

**Published:** 2017-04-19

**Authors:** A. K. L. Liu, H. M. Lai, R. C.‐C. Chang, S. M. Gentleman

**Affiliations:** ^1^ Neuropathology UnitDivision of Brain SciencesDepartment of MedicineImperial College LondonLondonUK; ^2^ Laboratory of Neurodegenerative DiseasesLKS Faculty of MedicineSchool of Biomedical SciencesThe University of Hong KongPokfulamHong Kong SAR; ^3^ State Key Laboratory of Brain and Cognitive SciencesThe University of Hong KongPokfulamHong Kong SAR; ^4^ LKS Faculty of MedicineResearch Centre of Heart, Brain, Hormone, and Healthy AgingThe University of Hong KongPokfulamHong Kong SAR

In recent years, advances in laser microscopy and endogenous fluorescent tagging techniques have led to the development of many tissue‐clearing strategies, which render tissues optically transparent, allowing large blocks of unsectioned tissue to be visualized in three dimensions (3D). CLARITY (Clear Lipid‐exchanged Acrylamide‐hybridized Rigid Imaging/Immunostaining/*In situ* hybridization‐compatible Tissue hYdrogel) is one of the tissue‐clearing techniques which works by fixation/hybridization of brain tissue using hydrogel cross‐links and subsequent detergent‐based delipidation to turn the tissue transparent 1. Since CLARITY enables molecular probing using immunofluorescence, this technique was deemed suitable for *post mortem* human brain tissues to demonstrate the potential in visualizing pathologies in Alzheimer's 2, Parkinson's 3 and neurodevelopmental disorders 1, 4 in 3D. Early attempts using CLARITY were performed on cortical tissue from the brains of children 1, 4, which is considered to be less myelinated than adults, or on thinly sectioned tissues of up to 500 μm in thickness 1, 2. In our previous study, we have demonstrated that CLARITY can successfully render larger blocks of tissue (about 3 mm in thickness) optically transparent in multiple cortical and subcortical regions of the human brain 3. However, we noticed the speed of tissue clearing differs between regions depending on the degree of myelination, and duration of formalin fixation. Densely myelinated regions such as the brainstem and spinal cord in archival formalin‐fixed tissues could not be rendered transparent with CLARITY. Also we, and other groups, have reported tissue expansion after tissue clearing with CLARITY 5. Although it was often claimed that the tissue expansion is a transient effect which will be adjusted by subsequent refractive index matching 6, the effects in human brain tissues, especially after prolonged (>40 days) passive tissue clearing, appeared to be irreversible 3. In addition, immunolabelling with antibodies, particularly on larger samples, remains challenging because the depth of antibody penetration is still limited 7. Many current existing tissue‐clearing protocols are now available and attempts have been made to improve tissue clearing by combining various protocols, such as CUBIC with RIMS in CLARITY and FRUIT (SeeDB with Scale) 8. Our aim was to develop an improved and simplified protocol for tissue clearing in the human brain.

The use of acrylamide hydrogel in the CLARITY protocol poses a number of problems including those described above. Tissues embedded with acrylamide hydrogel undergo expansion upon sodium dodecyl sulphate (SDS) clearing and become more fragile as structural integrity is lost 3, 5, 9. In addition, when transcardial perfusion cannot be performed, diffusion of hydrogel monomers within a large block of tissue may be limited, leading to incomplete tissue hydrogel hybridization 9. Furthermore, although pores in polyacrylamide matrices aid lipid exchange and antibody penetration can be enhanced by changing composition of the hydrogel 5, immunolabelling distance appeared to be better in formaldehyde‐fixed, unhybridized tissue (without acrylamide hydrogel) compared with those hybridized with both formaldehyde and acrylamide 6. The original CLARITY study suggested cross‐links between hydrogel and formaldehyde aid fixation of protein and nucleic acids during the delipidation process. Protein loss was reported to be significantly lower in acrylamide‐embedded tissues compared with unhybridized tissue after SDS clearing 1, 5, 6. However, there has been no evidence showing the existence of hydrogel–formaldehyde cross‐links. Also, in our recently published study, no significant protein loss was found after SDS clearing of formalin‐fixed human brain tissues 10. Besides, protein loss does not necessarily compromise the quality of immunostaining. Hence, as long as the tissue is well fixed in formaldehyde, we recommend the tissue‐clearing procedure to be simplified by omitting the use of acrylamide‐based hydrogel.

Prolonged formaldehyde fixation impedes clearing speed and immunolabelling due to excessive formaldehyde cross‐links on the tissue. One simple solution is to use fresh tissue instead of formalin‐fixed material. Densely myelinated regions such as brainstem, spinal cord and cortical white matter are difficult to render transparent with SDS delipidation 3. Reiner and colleagues introduced iDISCO which demonstrated that it is possible to immunostain a piece of optically opaque, formaldehyde‐fixed tissue by permeabilizing it in a cocktail of detergent 11. The tissue can then be rendered optically transparent with organic solvents using the 3DISCO clearing technique 12. Apart from its delipidation properties, SDS is known to be a protein denaturant, which accounts for its use for antigen retrieval in traditional immunohistochemistry. As a result, to improve immunolabelling in tissue clearing, the combination of SDS delipidation in CLARITY and detergent permeabilization in iDISCO can be useful. In addition, we observed that for densely expressed antigens, such as glial fibrillary acidic protein GFAP and neurofilament, the use of a low antibody concentration (1:1000) with daily supplement (to a concentration of 1:100–1:50) may be useful to prevent antibodies being ‘trapped’ at tissue surface.

With the omission of acrylamide–hydrogel in CLARITY and combination with the iDISCO technique, we introduce FASTClear (Free of Acrylamide SDS‐based Tissue Clearing) for the immunostaining and three‐dimensional visualization of human brain tissue (Table 1). First, fresh human brain blocks of up to 1 cm in thickness were fixed in 4% paraformaldehyde or 10% neutral‐buffered formalin at 4°C for 3 days. Then, the tissue was trimmed to about 3 mm in thickness (the maximum working distance of confocal objectives) before delipidation in 4% SDS‐boric acid buffer at 50°C for a minimum of 5 days. Best immunostaining was achieved if the region of interest is rendered optically transparent at this stage. However, it is possible to proceed to immunostaining even if the tissues do not achieve full transparency at this point. The tissue was then washed thoroughly in phosphate‐buffered saline (PBS) with 0.1% Triton‐X 100 (PBS‐Triton) at 50°C (3 × 1 h). Then, the tissue was permeabilized and blocked in 0.6 M glycine, 0.2% Triton X‐100, 6% Donkey serum, 20% dimethyl sulfoxide (DMSO) dissolved in PBS overnight at 37°C. Next, after washing the tissue in PBS‐Triton for 2 × 1 h at 37°C, it was incubated with primary antibody diluted in 0.2% Tween‐20, 5% DMSO, 3% Donkey serum, 0.01% sodium azide in PBS for a minimum of 2 days at 37°C. Following another wash in PBS‐Triton (3 × 1 h, then overnight incubation at 37°C), the tissue was incubated with a secondary antibody conjugated with Alexa Fluor^©^ fluorophores diluted in the same diluent as above for the same number of days as primary antibody incubation at 37°C. A nuclear counterstain, 4′,6‐diamidino‐2‐phenylindole (1:100 from a stock of 1 μg/ml diluted with 1:1 water: DMSO), can be added at this stage. After that, the tissue was washed thoroughly in PBS‐Triton (5 × 1 hr; then overnight) and proceeded to refractive index matching. For tissues that have been rendered optically transparent at the delipidation step, immersion in 47% 2,2′‐thiodiethanol (vol/vol) in 0.01 M PBS without saline or 70% w/v sorbitol in 0.1 M phosphate buffer as previously described 3 was done for refractive index matching. For tissues that did not achieve transparency at the delipidation step (or if microscope objectives are designed for high refractive index solution), they can be dehydrated and refractive index matched as per the 3DISCO protocol 12. Briefly, tissue was immersed in 50% tetrahydrofuran (THF) (overnight), 70% THF (1 h), 80% THF (1 h), 100% THF (1 h), 100% THF (1 h) and finally dibenzyl ether until the tissue became optically transparent. Tissues were then mounted and visualized using a single‐photon or two‐photon confocal microscope.

**Table 1:** Free of acrylamide sodium dodecyl sulphate (SDS)‐based tissue‐clearing (FASTClear) protocol

Step	Time
1. Fixation in 10% neutral‐buffered formalin/4% paraformaldehyde (PFA)	Minimum of 3 days at 4°C
• N.B. fixation time depends on size of fresh tissue block. Typically, a 1‐cm‐thick block will take around 3 days to be fully fixed.	
• This step is only required for fresh tissue. Proceed to Step 2 for formalin‐fixed tissue.	
Caution: use a tightly sealed container (±parafilm) for fixation as formalin/PFA is toxic.	
2. Dissect into smaller block	
• A maximum of 3 mm in thickness is recommended due to immunolabelling diffusion and confocal objectives working distance limits. Note that the sectioning surface should be as flat as possible and designed to be the future imaging surface.	
3. Immerse in 4% SDS buffer	Minimum of 5 days at 50°C oven
• This step improves antibody labelling and it is recommended the tissue is immersed in SDS buffer until transparency is reached. For prolonged fixed tissue (>2 years in fixation), a 2‐mm‐thick block can reach transparency in 3 months.•[However, do note that complete transparency of tissue is not a necessity as tissue will become transparent at the final refractive index matching step]	
• Frequent change in buffer (daily to twice weekly) can improve the speed of reaching transparency.	
4. Washing in 0.1% phosphate‐buffered saline (PBS)‐Triton	3 × 1 h at 50°C
5. Blocking and permeabilization in blocking medium [0.6 M glycine, 0.2% Triton X‐100, 6% Donkey serum, 20% dimethyl sulfoxide (DMSO) in PBS]	Overnight at 37°C
• Add enough blocking medium to cover the tissue.	
• Optional if the antibody is known to be of high specificity.	
6. Washing in 0.1% PBS‐Triton	2 x 1 h at 37°C
7. Primary antibody incubation (diluted in 0.2% Tween‐20, 5% DMSO, 3% Donkey serum, 0.01% sodium azide in PBS)	Minimum of 2 days at 37°C
• Start with a low concentration (e.g. 1:1000; 2 μl in 2 ml of diluent), supplementing antibody daily/twice daily until a final concentration of around 1:50–1:100 is reached.	
• Optimal concentration and days of incubation vary between antibodies.	
• As an example, tyrosine hydroxylase antibodies (Millipore AB152) can reach complete penetration to a depth of 2 mm on each side in 3 days at a final concentration of 1:100.	
• If multiple antigen labelling is required, it is recommended to perform immunolabelling sequentially.	
8. Washing in 0.1% PBS‐Triton	3 × 1 h at 37°C; then overnight at 37°C
9. Secondary antibody incubation (diluted in 0.2% Tween‐20, 5% DMSO, 3% Donkey serum, 0.01% sodium azide in PBS)	Minimum of 2 days at 37°C
• Same as Step 7 above.	
• 4′,6‐Diamidino‐2‐phenylindole or fluorophore‐conjugated lectin can be added at this stage (1:100 from a stock of 1 μg/ml diluted with 1:1 water: DMSO) for better tissue orientation.	
10. Washing in 0.1% PBS‐Triton	5 × 1 h at 37°C; then overnight at 37°C
11. Immersion in refractive index matching medium	RT until transparency is reached
• If tissue is transparent/almost transparent after Step 3	
Immerse in 47% 2,2′‐thiodiethanol diluted in 0.01 M PBS without saline or 70% w/v Sorbitol in 0.1 M phosphate buffer (as previously described in ref. 3).	
• If tissue is opaque after Step 3, follow 3DISCO clearing method:	
Dehydrate tissue in 50% tetrahydrofuran (THF) (overnight), 70% THF (1 h), 80% THF (1 h), 100% THF (1 h) 100% THF (1 h) then dibenzyl ether until transparency is reached.	
Caution: perform this step in the fume hood.	

We applied this protocol on a piece of fresh spinal cord tissue, which is difficult to render transparent with the traditional CLARITY technique, and successfully visualized the three‐dimensional structure of the ventral root to a depth of 508.52 μm using immunostaining for neurofilament (Figure 1 **a**). However, it has to be noted that there is heterogeneity in the penetration depth of the antibody with some parts of the spinal cord with labelling to 89 μm only. The reasons behind the variation in penetration depth within the same piece of tissue remain largely unknown, and we hypothesize that local tissue structural biochemical properties or vasculature can affect antibody penetration. Furthermore, we have also demonstrated the potential of FASTClear with other antibodies such as tyrosine hydroxylase (Millipore AB152, UK) and microtubule‐associated protein 2 (abcam ab5392, UK) (data not shown) and on formalin‐fixed tissue in a tissue bank for the 3D visualization of Purkinje neurons in the cerebellum (Figure 1 **b**), with a depth of immunolabelling to 66.5 μm.

**Figure 1:**
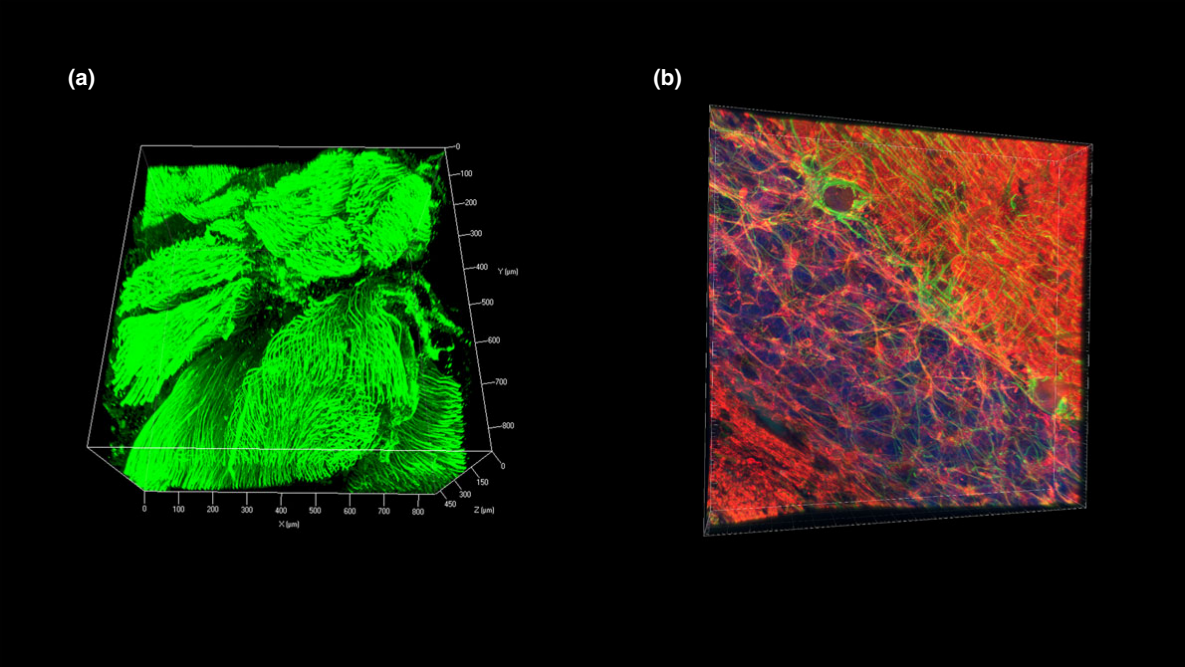
Human brain tissue processed and immunostained using free of acrylamide sodium dodecyl sulphate (SDS)‐based tissue clearing (FASTClear). (**a**) z‐Stack image of a ventral root of a piece of spinal cord fresh tissue (3 mm thick) immunostained using antineurofilament primary antibody (final concentration 1:100; Dako M0762) and Alexa‐fluor 488 conjugated donkey anti‐mouse secondary antibody. Stained tissue was visualized using a Zeiss 780 inverted confocal microscope (Carl Zeiss, Germany) with ×10 objective (imaging depth to 508.519 μm, z‐stack step size 3.03 μm). (**b**) z‐Stack image of a piece of fixed cerebellar tissue immunostained using antibodies against neurofilament (green; final concentration 1:100; Dako M0762) and βIII‐tubulin (red; final concentration 1:100; Millipore AB9354) and counterstained with 4′,6‐diamidino‐2‐phenylindole (blue). Stained tissue was visualized using a Leica SP5 (Leica Microsystems, UK) confocal microscope with ×40 objective (imaging depth to 66.5 μm, z‐stack step size 0.38 μm).

FASTClear is a greatly simplified and more user‐friendly tissue‐clearing protocol for human brain tissues which reduces the overall processing time from tissue fixation to immunostaining and visualization to a minimum of 16 days (Figure 2). However, it is still not possible to achieve immunolabelling to the full thickness of the tissue and this technique is yet to be optimized for archival formalin‐fixed tissues. Also, tissue that requires clearing with organic solvent may undergo shrinkage. Although fine structures are likely to be preserved, a high‐power objective will be required which often has a lower working distance. Nevertheless, with the development of smaller molecular probes such as nanobodies and aptamers 7, further improvement of human brain tissue clearing will be made, leading to a new era of three‐dimensional histology.

**Figure 2:**
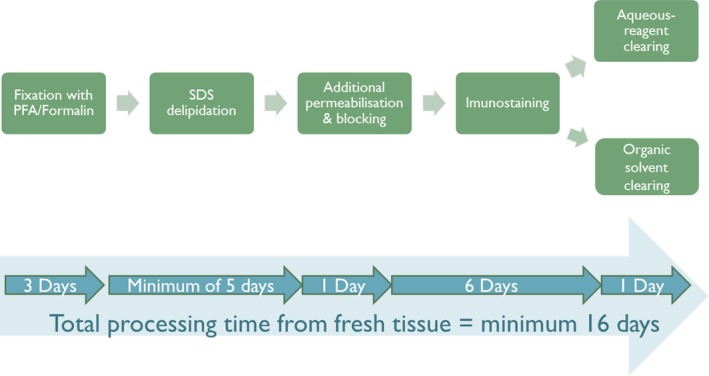
Workflow diagram of free of acrylamide sodium dodecyl sulphate (SDS)‐based tissue clearing (FASTClear).

## Author contributions

All authors contributed to the experimental design and conceived the study. A.K.L.L. and H.M.L. carried out all the experimental work, drafted and revised the manuscript. R.C.C.C. and S.M.G. supervised the research. All authors read, reviewed and edited the final manuscript.

## Ethical considerations

The work conducted on human tissue was under ethical approval held by the Parkinson's UK Brain Bank at Imperial College London [Registered charity in England and Wales (258197) and in Scotland (SC037554); Multicentre Research Ethics Committee approval reference number: 07/MRE09/72]. Parkinson's UK Brain Bank is an approved Research Tissue Bank by the Wales Research Ethics Committee (Ref. No. 08/MRE09/31+5). Informed consent was obtained prospectively for the use of *post mortem* brain tissues and brain samples were obtained and prepared in accordance to the Wales Research Ethics Committee approved protocols.

## Conflict of interest

All authors declare no conflict of interest.
